# PLGA/PLA-Based Long-Acting Injectable Depot Microspheres in Clinical Use: Production and Characterization Overview for Protein/Peptide Delivery

**DOI:** 10.3390/ijms22168884

**Published:** 2021-08-18

**Authors:** Arun Butreddy, Rajendra Prasad Gaddam, Nagavendra Kommineni, Narendar Dudhipala, Chandrashekhar Voshavar

**Affiliations:** 1 Formulation R&D, Biological E. Limited, IKP Knowledge Park, Shameerpet, Hyderabad 500078, India; butreddyarun@gmail.com (A.B.); prasadmpharm@gmail.com (R.P.G.); 2 Department of Pharmaceutics, National Institute of Pharmaceutical Education and Research, Hyderabad 500037, India; nagavendra.kommineni@gmail.com; 3 Department of Pharmaceutics, Vaagdevi College of Pharmacy, Warangal 506005, India; 4 College of Pharmacy and Pharmaceutical Sciences-Institute of Public Health, Florida A&M University, Tallahassee, FL 32307, USA

**Keywords:** PLGA, PLA, proteins/peptides, microspheres, manufacturing techniques, characterization techniques

## Abstract

Over the past few decades, long acting injectable (LAI) depots of polylactide-co-glycolide (PLGA) or polylactic acid (PLA) based microspheres have been developed for controlled drug delivery to reduce dosing frequency and to improve the therapeutic effects. Biopharmaceuticals such as proteins and peptides are encapsulated in the microspheres to increase their bioavailability and provide a long release period (days or months) with constant drug plasma concentration. The biodegradable and biocompatible properties of PLGA/PLA polymers, including but not limited to molecular weight, end group, lactide to glycolide ratio, and minor manufacturing changes, could greatly affect the quality attributes of microsphere formulations such as release profile, size, encapsulation efficiency, and bioactivity of biopharmaceuticals. Besides, the encapsulated proteins/peptides are susceptible to harsh processing conditions associated with microsphere fabrication methods, including exposure to organic solvent, shear stress, and temperature fluctuations. The protein/peptide containing LAI microspheres in clinical use is typically prepared by double emulsion, coacervation, and spray drying techniques. The purpose of this review is to provide an overview of the formulation attributes and conventional manufacturing techniques of LAI microspheres that are currently in clinical use for protein/peptides. Furthermore, the physicochemical characteristics of the microsphere formulations are deliberated.

## 1. Introduction

Protein and peptide drugs are known to be some of the most effective therapies to elicit a desired therapeutic activity due to their specific interactions with biological targets [1]. However, effective administration of protein/peptide drugs require repeated doses as these drugs exhibit low half-lives and are rapidly cleared from systemic circulation [2]. In addition, repeated dosing or administration leads to low patient compliance affecting the overall effectiveness of the protein/peptide therapeutics [3]. When administered orally, protein therapeutics show poor bioavailability, due to rapid enzymatic degradation in the gastrointestinal tract, and restricted membrane permeability, limiting these drugs to parenteral (intravenous (IV), subcutaneous (SC), and intramuscular (IM) injections) route administration [4]. Polymer microparticle is a particle of polymer of any shape with an equivalent diameter of approximately 0.1 to 100 μm [5]. They are made up of natural or biodegradable polymeric materials that entrap or encapsulate proteins/peptides or other biologically active substances. In general, microspheres can be suspended in an aqueous vehicle and delivered parenterally with a tiny gauge needle without anesthesia [6]. The particles either feature a continuous polymeric matrix with uniform drug dispersion or a shell-like wall around the drug reservoir/core [6]. Microspheres have a number of advantages over conventional controlled drug delivery systems, such as (1) ability to customize the rate and duration of drug release by changing the materials and manufacturing procedures; (2) microspheres are more stable than alternative controlled drug delivery technologies, such as liposomes; and (3) patient compliance is improved because of the shorter dose frequency [6,7,8].

Long-acting injectable (LAI) microspheres have been previously exploited for the delivery of protein and peptide therapeutics owing to their high drug loading capacity and potential to provide prolonged drug release for extended periods. The advantages of LAI microspheres include enhanced stability and bioavailability and improved efficiency and patient compliance [9]. PLGA/PLA-based LAI microspheres could have a positive impact on the delivery of proteins/peptides because: PLGA microspheres can be used as a solution to the inconvenience and discomfort of frequent injections; by enabling the delivery of drugs to targeted areas, higher drug concentrations can be maintained in the targeted area, thus reducing systemic exposure; they may improve treatment adherence, reducing relapse frequency and rehospitalization rates; they could reduce the risk of accidental or deliberated dose; they allow for the ability to treat patients with more stable plasma concentrations than oral medications; avoiding first-pass metabolism means there is a better relationship between dose and blood level of drug, meaning lower and less frequent peak plasma, which reduces side effects [10,11]. The target product profile of LAI microspheres contains a number of formulations and process development variables, which include: developing a formulation that provides a minimal burst release and acceptable plasma concentration; selecting a polymer type that provides the required duration of drug release while maintaining the stability of proteins/peptides during storage and in vivo; developing a suitable and reproducible process that is scalable to commercial production. The two main challenges in the preparation of LAI microspheres that impede their development into a commercial success are; (1) achieving uniform size distribution of microspheres at large scale production; (2) consistent bioactivity of encapsulated drug during preparation, storage, and release as they are subjected to various forces such as shear stress (during homogenization), oil–water interface stress, and ice–liquid and dehydration stress (during lyophilization), leading to significant loss of their therapeutic effect [12,13,14,15,16]. The ideal microsphere formulation should have reasonably high protein loading capacity, encapsulation efficiency, and sustained release potential of encapsulated protein while retaining its biological activity [8]. In addition, the particle size of the microspheres formulation should be small enough to pass through a needle of 22–25 gauge for IM and SC administration [17].

Protein and peptide therapeutics have been encapsulated in polymer matrixes to create microspheres or micro particles. Biocompatible and biodegradable polymers such as polylactide-co-glycolide (PLGA) and polylactic acid (PLA) are most commonly employed and widely utilized at a clinical level. The Food and Drug Administration (FDA) have approved a number of LAI microsphere formulations because of their proven safety history. Details of some commercially available PLGA/PLA-based microsphere formulations are presented in Table 1.

Although currently available commercial PLGA-based LAI microspheres have often shown success, there are several challenges that limit the broader development and application of LAI microspheres. Peptide and protein instability, poor release kinetics of PLGA, presence of residual solvents in the product, and increased cost of manufacturing are the major issues [13,20,21]. Despite several PLGA-based LAI microspheres being marketed well beyond their patent expiration, there are currently no generic PLGA-LAI microspheres approved by the FDA. This could be related to the formulation and manufacturing difficulties related to LAI microspheres having hampered the progress of generic product development [22]. Because PLGA is a significant element in LAI microspheres, its composition and amount are critical for the microsphere drug release. The first stage in the development of LAI microspheres is to identify a PLGA polymer. Understanding and managing drug release kinetics from PLGA-based microspheres requires a thorough analysis of PLGA, such as the lactic acid (LA)/glycolic acid ratio (GA) (LA/GA), molecular weight and distribution, and polymer end group [23,24]. There are several factors that influence the release profile of protein/peptides from PLGA microspheres, particularly during the manufacturing process [25]. The solvent and emulsifier used in the preparation, the drug distribution in the microsphere, the microsphere’s apparent and intrinsic properties, and the in vitro testing method all play a significant role in the release behavior and, as a result, the product performance of microspheres [26]. Furthermore, minor changes in manufacturing techniques can alter the physicochemical properties of the microspheres [27]. Therefore, in the development of LAI PLGA/PLA-based microspheres, elucidation of quality attributes and essential manufacturing process parameters is a major concern. The present review discusses the key formulation attributes, and the manufacturing techniques employed for the development of PLGA/PLA-based LAI microspheres for protein/peptide delivery. Furthermore, important physicochemical properties of protein/peptide loaded LAI microsphere formulations are discussed.

## 2. Formulation Attributes of PLGA/PLA-Based LAI Microspheres

### 2.1. PLGA/PLA Polymer

**Figure 1:**
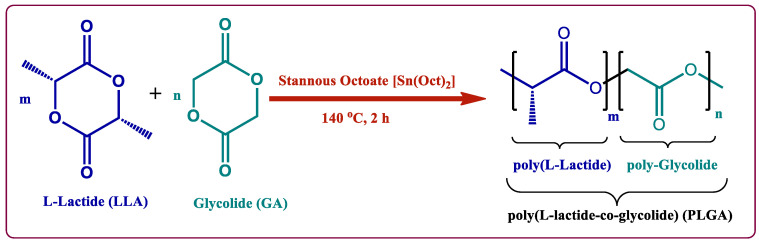
Schematic representation of the synthesis of poly(lactide-co-glycolide) (PLGA: m = number of lactide units, and n = number of glycolide units).

The selection of the PLGA/PLA polymer for the development of microspheres depends on the route of administration, which is specific for a particular drug, the amount of microspheres administered per unit dose, rate of drug release from the microspheres in a day to meet the therapeutic concentration of the specific drug, and the degradation time of the polymer. Although the drug release from the microspheres is faster than the complete degradation of the polymer, the degradation time of the polymer plays a major role in the selection of the suitable polymer [28]. PLGA is the most studied biodegradable polymer for commercial and experimental drug encapsulation, as they are biocompatible and degrade into non-toxic oligomers or monomers. It is commercially available in different copolymer compositions, capping groups, and molecular weight(s) (MW), which offers possibilities to tune drug release kinetics and degradation. A PLGA polymer is synthesized by ring-opening polymerization of lactide and glycolide monomers, using stannous octoate as a catalyst, which activates the hydroxyl moieties to initiate ring-opening polymerization [29]. Ring opening polymerization of cyclic monomers, as L-lactide (LLA) and glycolide (GA), is the most widely applied method for the synthesis of PLGA copolymers with an appropriate catalyst such as stannous octoate ([Sn(Oct)2]). The [Sn(Oct)2] is used to open the lactide and glycolide rings because it is a highly efficient transesterification agent. Its typical bulk reaction times at 100–180 °C ranges from a few hours to days, resulting in copolymers with a random microstructure [30,31]. Figure 1 shows a schematic representation of the synthesis of poly(lactide-co-glycolide) (PLGA).

**Figure 2:**
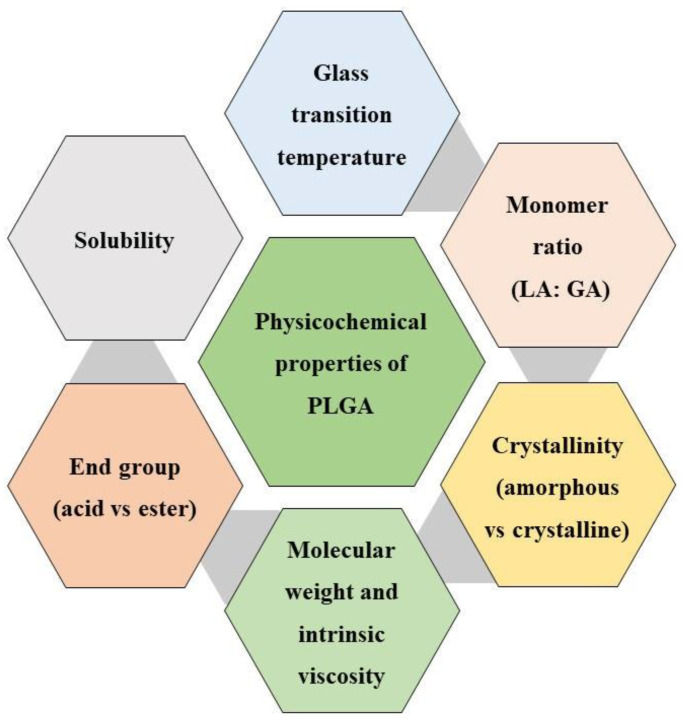
Some key physicochemical properties of the PLGA polymer.

The hydroxyl moiety is usually attached to the growing PLGA chain via an ester bond. In the formation of PLGA with a free acid end-cap, water itself serves as an initiator [32]. Other alkyl hydroxyl initiators, such as dodecanol, forms PLGAs alkyl ester [33]. By controlling the access to hydroxyl group initiation sites, a wide arrangement of PLGA polymers can be synthesized [33]. The high quality PLGA can be obtained by homopoly- merization of methylglycolide and copolymerization of methylglycolide and glycolide [34]. The important physicochemical characteristics of the PLGA polymer in developing LAI microspheres are outlined in Figure 2. PLGA is composed of hydroxyl acid monomers (d-lactic, l-lactic and/or glycolic acids). By changing the molecular mass, monomer ratio, and end group chemistry, a PLGA polymer can encapsulate the different molecules of any size. In the development of LAI microspheres, understanding the physicochemical properties of the PLGA/PLA helps in achieving the target product profile. It is important to characterize the raw PLGA material and a PLGA present in the microsphere formulation because properties of the PLGA influence the final structures and other events during the microsphere formation. Typically, a complete characterization of PLGA relies on the measurements of the lactide to glycolide (L:G) ratio, MW, polymer shape (linear or branched), and end group (acid or ester) [35,36]. PLGA is a linear copolymer consisting of lactic acid and glycolic acid monomers, which is available in molecular weight ranges from below 10,000 to 200,000 g/mol [37]. As the molecular weight is directly related to the polymer degradation rate, the intrinsic viscosity of PLGA polymer is one crucial attribute directly related to the polymer molecular weight. The PLGA polymer with high inherent viscosity enhances the encapsulation efficiency of proteins/peptides by decreasing the tendency of protein molecules to diffuse out of the polymer matrix [35,38].

The selection of PLGA in the microsphere development depends on the appropriate selection of PLA and PGA ratio. This will determine the degradation kinetics of the microsphere formulation in the body. The hydrophilicity/hydrophobicity nature of the PLGA matrix depends on the lactide to glycolide ratio. PLA is hydrophobic in nature, whereas PGA is more hydrophilic; hence, PLGAs with a high ratio of PGA has a faster degradation rate owing to their hydrophilic nature, which promotes the absorption or penetration of water molecules into PLGA matrix, causing hydrolysis of polymer chains [39]. The higher the content of lactic acid monomer in the copolymer, the slower the degradation rate.

PLGA polymers with different lactide to glycolide ratios (e.g., PLGA 50:50 and PLGA 75:25) can exhibit different drug loading and drug release profiles owing to changes in their solubility profiles, degradation characteristics, and interactions between the drug and polymer [36]. The nature (crystalline or amorphous) of PLGA impact the encapsulation efficiency and degradation rate of the polymeric chain. The crystallinity of PLGA is influenced by the stereochemistry of the lactic acids and L:G molar ratio. In addition, copolymers composed of L-PLA and PGA are semi-crystalline, whereas copolymers prepared with D, L-PLA and PGA are amorphous. D, L-PLGA is preferable in microsphere formulations because the encapsulated drug molecules are dispersed more homogeneously in the amorphous polymer than the semi-crystalline one [40]. The PLGA polymer with amorphous domains degrades faster than the crystalline region because the amorphous domains are more accessible to water compared to crystalline ones, thus the degradation proceeds faster. Furthermore, the encapsulation efficiency increases with an increase in the amorphousness of the PLGA polymer [41].

The glass transition temperature (T_g_) is one of the important physicochemical properties of the PLGA polymer. The T_g_ of PLGA usually lies above the physiological temperature (37 °C) and increases with the lactide content and molecular weight of the polymer or the addition of compounds such as active pharmaceutical ingredients (API) or plasticizers [42,43]. The end terminal (acid or ester) group of PLGA is another important physicochemical parameter that directly affects the functionality of the microsphere formulation. The PLGA polymer with an ester end cap has shown a four to six week delay in in vivo degradation compared with the acid end group of PLGA with a similar monomer ratio and molecular weight [44]. Also, the presence of an acid end group in PLGA polymer causes the swelling of the PLGA matrix and initiates hydrolysis owing to its increased water uptake potential [44].

Generally, PLGA polymer is degraded into oligomers and monomers via hydrolytic scission of the ester bonds. The degradation and erosion of PLGA polymer can be explained in two stages [45]. In the first stage, a large amount of lactic and glycolic acids are formed resulting in decreased pH and under such conditions proteins/peptides encapsulated in PLGA are relatively stable. In the second stage, microspheres lose mass as a result of an increased polymer chain scission. In small particle sized microspheres, the degradation of PLGA is mostly homogeneous, whereas, in large particle sized microspheres, hydrolytic degradation is heterogeneous. During the degradation process, acid autocatalysis enhances the PLGA degradation rate due to an increase in the number of carboxylic end groups. The accumulation of acidic by-products can affect the PLGA microsphere porosity and internal structure [46,47]. Scission of long polymer chains causes a reduction in the polymer molecular weight, leading to an increase in its hydrophilicity and the formation of a water-soluble fragment. PLGA can also undergo auto-catalytic degradation, where an acidic by-product remains strapped in the bulk of the polymer, auto-catalyzing the degradation process and leading to the generation of a highly acidic microenvironment [32]. The formation of an acidic microenvironment depends on the nature of drugs and excipients embedded in the microspheres. The interaction of basic molecules with the PLGA polymer may either accelerate or decelerate the degradation. For instance, basic molecules may serve as catalysts to the ester bond cleavage that increases the degradation rate of the polymer [48]. In some cases, it is possible that basic molecules may protect the polymer terminal carboxylic residues and decrease the catalytic effect of the acidic end chains on polymer degradation [49,50]. When used in injectable microspheres, the degradation mechanism of PLGA polymer is as follows; polymer surface erosion with the release of the encapsulated drug; scission of polymer–drug bonding, and release of physically entrapped drug by diffusion [51]. Factors that can influence the degradation of PLGA include; polymer hydrophilicity or hydrophobicity, water permeability, monomer ratio (L:G ratio), MW, Tg, morphology (crystalline/amorphous), and pH [32].

Most of the injectable PLGA microsphere formulations in clinical use utilize a linear PLGA except Sandostatin^®^, which utilizes the star-shaped or branched PLGA, more specifically a glucose core with attached PLGA chains as glucose-initiated PLGA (Glu-PLGA) [36]. The number of branches (or arms) of the Glu-PLGA is a critical characteristic of the polymer. There has been limited information available on characterizing the molecular structure of Glu-PLGA. An accurate calculation of the branch units in Glu-PLGA depends on selecting the appropriate linear PLGA comparators that cover the entire molecular range of the branched PLGAs. Also, it is important to use PLGA of the same end cap and L:G ratio [52]. Linear PLGA polymers can easily be characterized by determining MW, end-cap, and L:G ratio. For star-shaped PLGA, determining the branch numbers and measuring the MW is not possible with the traditional methods unless a series of branched PLGA standards were used. Typically, Glu-PLGA polymer exhibits a faster mass loss and degradation rate than the linear PLGA of similar molecular weight. [36].

### 2.2. Organic Solvent

**Figure 3:**
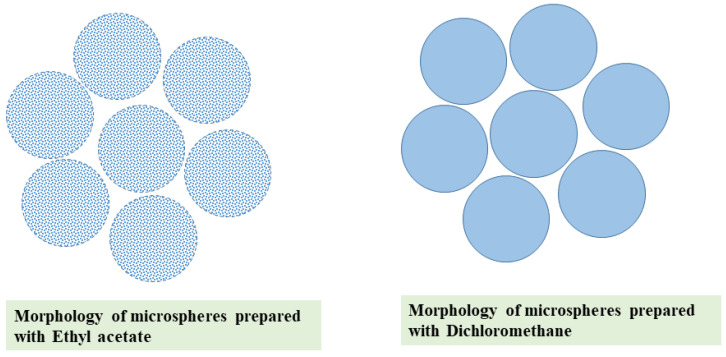
Schematic illustration of the particle morphology of microspheres prepared using ethyl acetate and dichloromethane.

Organic solvents are used to dissolve both the drug and the polymer in emulsion-based microencapsulation techniques. For efficient encapsulation, the organic solvent should meet the following criteria; low toxicity, low boiling point, high volatility, and ability to dissolve the polymer [53]. Methylene chloride is the most commonly used solvent for encapsulation owing to its low boiling point, high volatility, and immiscibility with water. In addition, high saturated vapor pressure of methylene chloride causes high solvent evaporation rate, which reduces the manufacturing process duration of microspheres [54]. However, the carcinogenicity property of methylene chloride limits its use in the fabrication of microspheres [55]. Ethyl acetate exhibits less toxicity as compared to methylene chloride and can be used as an alternate solvent. However, its partial solubility in water or miscibility with water limits the microsphere formation. The type of solvent on the microsphere morphology is shown in Figure 3. It has been observed that when dichloromethane is used as the solvent, the microparticles are spherical with a rough surface, while the microparticles have a smoother surface and micropores when ethyl acetate was used as solvent [53,56]. The difference in morphology (rough and smooth surface) could be due to the solvent evaporation rate. Solvent evaporation of dichloromethane is faster than that of ethyl acetate. The ability of the organic solvent to diffuse seems to be a key factor for the possibility of coalescence phenomena during polymer precipitation and particle formation [57]. When the dispersed phase (PLGA in ethyl acetate) is introduced directly into the continuous or aqueous phase, a sudden extraction of a large quantity of ethyl acetate from the dispersed phase leading to precipitation of the polymer as an aggregate [58]. However, pre-saturation of aqueous solution with an organic solvent such as ethyl acetate can resolve this issue by delaying extraction of the solvent and hardening the droplet surface [59]. Pre-saturation of continuous phase to a certain extent with ethyl acetate can control the particle size of the microspheres by altering the interfacial tension between the continuous and discontinuous phase, reduce the diffusion of ethyl acetate from the dispersed phase into the aqueous phase by decreasing the driving force for the solvent extraction, and prevent early precipitation of the PLGA (which helps in the formation of spherical microspheres instead of undesirable large clumps of drug–PLGA) [60,61]. The L:G ratio has a more significant impact on the polymer’s solubility in an organic solvent. As the lactide portion increases in L:G ratio, PLGA polymer become soluble in a large number of solvents. When the glycolide portion increases, PLGA dissolves only in highly fluorinated solvents, such as hexafluoroisopropanol [35].

Previous studies reported that the microspheres prepared with methylene chloride are more uniform and spherical, while the ethyl acetate-based microspheres appear to be partially collapsed. Moreover, encapsulation efficiency is high for microspheres made with methylene chloride than the microspheres prepared with ethyl acetate as a solvent [62,63]. The low encapsulation efficiency of ethyl acetate based microspheres could be due to more drug being bound to the continuous phase by the higher mass flux of solvent diffused from the dispersed phase into the continuous phase or the large quantity of solvent available in the continuous phase, which increases the solubility of the drug in the continuous phase, encouraging the diffusion of the drug into the continuous phase, and leading to the loss of drug [53]. In summary, solubility of polymer solvent in the continuous phase, the solubility of water in the polymer phase, solvent removal rate, and solvent toxicity and regulatory considerations are the key characteristics of the solvent that strongly influence the microsphere formation. During microsphere formulation preparation, the aqueous solubility or miscibility of the organic solvent will affect its initial extraction. Typically, a rapid precipitation of the polymer due to the initial extraction of the solvent to the external phase is beneficial for obtaining high encapsulation efficiency. Nevertheless, if a large volume of water is used or if the solvent is too soluble in the water, which results in fast solidification of the polymer, creating a dense polymer shell on the droplet forms a particle with a hollow core. The fast flux of the solvent out of the organic phase further disrupts the droplets and forms a smaller microsphere as the emulsion droplets shrink [64,65]. The solubility of the water in the organic phase will impact the reverse flux of the continuous phase into the dispersed phase, and thus, the porosity of the microspheres.

#### 2.2.1. Solvent Removal Rate

Depending on the boiling point and vapor pressure of the particular solvent, the solvent removal rate also relies on the evaporation of the solvent from the hardening batch, which is controlled by the unstirred boundary layer in the liquid or gas. The volatility of solvent can also impact the solvent removal rate from the microparticles. For a solvent with lower volatility, the solvent removal process can be expedited by increasing the temperature or applying reduced pressure. However, increasing the temperature of the hardening batch may lead to a more rapid flux of the solvent across the oil-in-water (O/W) interface, causing small fractures on the particles, results in increased porosity and lower drug loading [66,67]. For appropriate solvent evaporation rates at the industrial level, a frequent replacement of the gas phase in the closed vessel by effectively flushing the liquid surface is recommended [28,68].

#### 2.2.2. Solvent Toxicity and Regulatory Considerations

The toxicity of the solvent is crucial for the regulatory approval of the microsphere product. The maximum residual solvent level in the microsphere product depends on the toxicity of the respective solvent, and their maximum recommended levels are specified in the International Council for Harmonization (ICH) guidelines. Several methods, including elevated temperature drying or final lyophilization of the microspheres, help to reduce the final residual solvent levels to an acceptable value [28].

### 2.3. Stabilizer

During microsphere formation, an emulsifier or stabilizer is required to ensure the stability of the emulsion droplet until enough solvent has been evaporated/extracted (i.e., polymer concentration becomes high enough) from the oil droplet to maintain particle formation [69]. Typically, polyvinyl alcohol (PVA) is the most commonly used emulsifier or stabilizer to formulate PLGA microspheres. PVA is a semi-crystalline synthetic polymer, soluble in water and insoluble in other organic solvents. PVA is commercially available in different grades depending on the degree of hydrolysis and viscosity. Partially hydrolyzed PVA grades are available in the range from 84.2 to 89.0% (viscosity, 3.4 to 52.0 mPa.s), moderately hydrolyzed grades range from 92.5 to 96.5% (viscosity, 14.5 to 30.0 mPa.s), and fully hydrolyzed grades range from 98.0 to 99.0% (viscosity, 4.0 to 60.0 mPa.s) [70]. The partially hydrolyzed PVA grades contain residual acetate groups, which reduce the degree of crystallinity, providing greater aqueous solubility and increasing its potential to adhere to hydrophobic surfaces [71]. In contrast, a fully hydrolyzed PVA grade has a high degree of crystallinity, low aqueous solubility, and increases its ability to adhere to hydrophilic surfaces [71]. In PLGA-based microspheres, PVA is usually added in the external aqueous phase to stabilize the emulsion formed between the aqueous and organic phases. The hydroxyl groups present in the PVA will interact with the water in the aqueous phase. In contrast, the vinyl chain of PVA will interact with the organic phase (dichloromethane; DCM) and remain trapped in the polymeric matrix, thus making the emulsion formed more stable. Variation in concentration and volume of the PVA solution will affect the emulsion stability [72]. During manufacturing, the presence of PVA reduces the interfacial tension between the two liquid phases and prevents the coalescence of the droplets. The particle size and distribution of microspheres greatly depend on the concentration of the PVA in the emulsion system [73]. Despite the repeated washing, the fraction or residual of PVA remains associated with the microsphere particles because PVA forms an interconnected network with the polymer at the interface. The binding of PVA to the microparticle surface is likely to happen when the organic solvent is evaporated or removed from the interface in which interpenetration of the PVA and PLGA molecules occurs. The residual amount of PVA associated with the microspheres can influence the properties of the microspheres, including particle size, protein loading, and in vitro release of the encapsulated protein [74]. Various factors that could affect the amount of residual PVA include the organic solvent used to prepare the polymer solution a d the concentration of the PVA in the continuous phase as well as the external aqueous phase.

The polarity of the organic solvent used in the emulsion system can affect the amount of PVA adsorbed at the interface (polymer–organic solvent–water). With the increasing organic solvent miscibility in water, the residual PVA associated with the microspheres increases. This is due to a higher amount of PVA being portioned into the polymeric phase containing an organic solvent, which is more miscible in the aqueous phase, causing the deposition of the higher portion of PVA on the surface of the microspheres [74].

#### PVA Concentration

Drug loading into PLGA microspheres depends on the PVA concentration. The lower the concentration of PVA, the lower the wettability of formed microspheres will be. There is a possibility that a lower concentration of PVA might not be high enough to create a stable emulsion due to leakage of the drug before skin formation of the microparticles. Chitkara and Kumar et al. evaluated three different concentrations (0.5%, 1%, and 2% *w*/*v*) of PVA in the bovine serum albumin-PLGA nanoparticles made by the water-in-oil-in-water (W/O/W) double emulsion technique. The highest encapsulation efficiency was obtained at 1% *w*/*v* PVA concentration [75]. The particle size of microspheres is likely to be dependent on the concentration of PVA in the external aqueous phase, and the smaller microsphere particle size was obtained from the higher PVA concentrations [72]. The higher PVA concentration in the continuous phase increases the density of PVA molecules at the O/W interface of the emulsion droplet, resulting in increasing the thickness of PVA on the droplet surface. The content of PVA per weight of microspheres increases with a decrease in particle size as the specific surface area decreases. When the concentration of PVA is increased, or the molecular weight/viscosity of PVA is increased, it prevents the separation of nascent emulsion droplets and increases the particle size of the microspheres, leading to the aggregation or coalescence of particles [76,77].

## 3. Manufacturing Techniques

**Figure 4:**
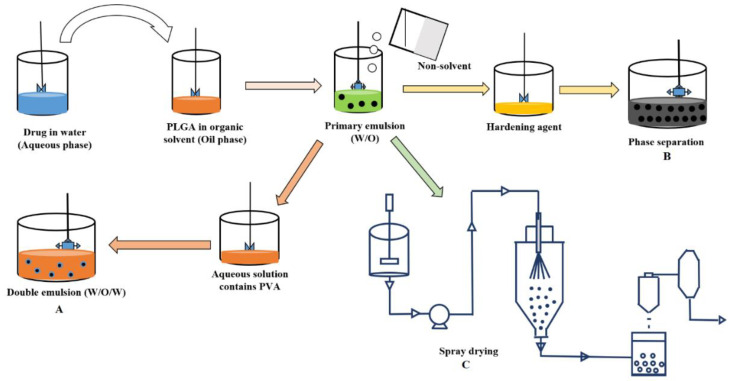
Schematic representation of conventional manufacturing techniques for the development LAI depots of PLGA/PLA microspheres for protein/peptide delivery. (**A**) emulsion–solvent evaporation, (**B**) coacervation/phase separation, and (**C**) spray drying.

The manufacturing techniques for the preparation of microspheres for parenteral delivery of proteins or peptides have a great impact on the resulting microsphere properties such as particle size, porosity, and surface morphology. Furthermore, the manufacturing technique should be well controlled and easy to scale-up. For the preparation of protein or peptide loaded PLGA/PLA microspheres, the most widely used conventional techniques are described below, which include double emulsion solvent evaporation, phase separation-coacervation, and spray drying (Figure 4).

### 3.1. Emulsification-Solvent Evaporation

The microencapsulation of PLGA/PLA-based drug products via solvent evaporation/extraction usually involves the formation of a single emulsion (O/W) or double emulsion (W/O/W). For poorly soluble molecules, the O/W method is frequently used. This method has the following steps: dissolution of the insoluble drug in an organic solvent containing the PLGA or PLA polymer; emulsification of organic or dispersed phase in an aqueous or continuous phase; extraction of the solvent from the dispersed phase by the continuous phase, accompanied by solvent evaporation, converting the droplets of the dispersed phase into solid particles; and then collection and drying of microspheres to eliminate the residual solvent [78]. This O/W method is not suitable for encapsulation of highly hydrophilic drugs due to the fact that hydrophilic drugs during emulsion formation diffuse into the continuous phase, leading to loss of drug. The O/W emulsion is produced by agitation or homogenization of two different phases, and the agitation process is continued until the solvent is partitioned into the aqueous phase and is subsequently removed by evaporation [54].

In general, organic solvents can be removed from the emulsion system by evaporation to a gas phase or by extraction into the continuous phase. For solvent evaporation, the carrier solvent must be dissolved in the continuous phase before evaporation occurs.

#### Double Emulsion Solvent Evaporation

The W/O/W double emulsion method is suitable for encapsulating hydrophilic molecules, and the main steps include the formation of primary and secondary emulsions and the removal of organic solvents using a suitable washing/evaporation process. To form a primary emulsion, an aqueous solution containing hydrophilic molecule is added to the solution of polymer previously dissolved in the water-immiscible organic solvent such as methylene chloride, chloroform, ethyl acetate, and then the mixture is emulsified by vortex, homogenization or ultrasonication [79,80]. PVA as an aqueous emulsifier is added to the primary emulsion to prevent aggregation of droplets in the double emulsion system. The double emulsion formed is transferred to an aqueous medium to evaporate the organic solvent and harden the microspheres. Then, the excessive PVA solution or unloaded drug is removed from the formed microspheres by washing the microspheres with water and then lyophilizing it for storage [72,81]. In the solvent extraction process, the emulsion is transferred into a large volume of quench or wash solvent medium, and the solvent associated with the oil droplets is diffused out. Meanwhile, in the evaporation process, the emulsion is exposed to a large quantity of water or co-solvent under appropriate temperature or pressure [45]. The double emulsion method is capable of producing microspheres with efficient encapsulation, high yield, and suitable for temperature-sensitive drugs [82,83]. However, leakage of water soluble drugs from the polymer phase to the outer aqueous phase may limit the encapsulation of the hydrophilic drugs [80]. The solvent removal step from the emulsion particles is one of the most critical factors in the double emulsion method because water-in-oil (W/O) emulsion droplets are exposed to a large quantity of water to remove the solvent and harden the microspheres. As the solvent is removed from the emulsion droplets into the aqueous media, the encapsulated protein molecules may diffuse from the emulsion into the aqueous media, accumulating on the microparticle surface as they become hardened, leading to a lower loading capacity and encapsulation efficiency and a higher initial burst release [17].

The stability of the primary emulsion is crucial for the successful encapsulation of proteins or peptides in the double emulsion technique. Schugenes et al. [84] studied the influence of emulsion stability on the characteristics of the microspheres (morphology and porosity) prepared by the W/O/W double emulsion technique using two different molecular weights of semi-crystalline L-polylactide polymer. The high viscosity of the polymer solution due to the increase in molecular weight of L-polylactide polymer leads to a less stable primary emulsion and more porous microspheres. The functional properties of microspheres can be tailored by changing several parameters, including; polymer, emulsifier, the organic solvent used, drug to polymer ratio, and the parameters of the emulsification, extraction, or evaporation process [45].

### 3.2. Coacervation

Coacervation or phase separation is a process by which the polymer solution is separated into two immiscible equilibrium liquid phases: dense coacervate phase concentrated in polymer and a dilute (supernatant liquid) polymer phase. The coacervation technique relies on the decreasing solubility of the coating polymer with the addition of a third component (coacervating agent) to the polymer solution in an organic solvent [85]. Coacervation can be initiated by a change in ionic strength, change in temperature, or addition of a non-solvent. These changes promote polymer–polymer interactions rather than polymer–solvent interactions, resulting in the dehydration of the polymer [85,86,87,88]. The main steps in microencapsulation by the coacervation technique includes the phase separation of the coating polymer solution, adsorption of the coacervate around the protein molecules, and solidification of the microparticles [85].

Generally, the polymer separation or formation of the polymer-rich phase is induced by adding a coacervating agent, typically silicone oil. Depending on the polymer molecular weight, concentration, and processing temperature, the phase separation occurs above the critical concentration of the silicone oil. The forming coacervate droplets are hardened by adding a hardening agent such as hexane or octamethylcyclotetrasiloxane. The main drawback associated with this technique is residual coacervating or hardening agent present in the microspheres, which may cause a problem of reduced biocompatibility [89]. Briefly, the coacervation process consists of [90]: (i) formation of W/O emulsion by dispersing the aqueous protein/peptide solution into a polymer dissolved organic phase (DCM) using suitable methods such as homogenization and sonication; (ii) formation of coacervate by gradually adding silicone oil (coacervate agent) to promote the phase separation, at which point the DCM is extracted into the silicone oil phase, causing embryonic microspheres to begin to precipitate; (iii) transfer of the mixture into a medium (heptane) to remove the solvents and harden the soft microspheres; (iv) the excess solvents will be removed by washing the microspheres with water, and the collected microspheres are sieved and dried under suitable conditions. In the coacervation method, the PLGA/PLA polymer acts as a wall or coating polymer, which deposits on the surface of the drug to achieve good encapsulation while the coacervate agent works as the phase inducer [91]. The incompatibility between the polymer and the coacervation agent is mainly responsible for the induction of phase separation [23].

### 3.3. Spray Drying

The preparation of microspheres by spray drying has been reported for the encapsulation of proteins to improve the stability of proteins. The spray drying method can overcome the problem of large volumes of solvent contaminated water phase associated with the emulsion based microencapsulation methods. However, spray-drying requires relatively large batch sizes compared to emulsion methods. Therefore, spray drying is often less suitable in the early stage development of microsphere formulation [28]. The spray drying process can be divided into (i) atomization of the liquid feed into droplets through an atomizer by transferring the emulsion solution through tubing at a certain speed into the atomizer; (ii) drying of the atomized droplets once the sprayed droplets and dry heated airflow enter the drying chamber, where the mixing of atomized droplets as well as drying medium (nitrogen gas) occurs; (iii) solvent evaporation via heat transfer from the drying medium to the droplets and the mass transfer of vaporized moisture from the droplets into the air allows for fast evaporation of the moisture and subsequent particle formation; and (iv) separation of the dried particles from the drying medium using a cyclone separation or baghouse filtration [92]. The air and the dried particles enter the cyclone tangentially, and the air will follow a strong vortex motion, forming a spiral pattern movement. For the particles with higher density or larger size, it is hard to follow the air stream; thus, the particles will strike the glass wall and fall into the collection vessel owing to the centrifugal forces [93].

In the spray drying method, the aqueous solution of proteins or peptides is dispersed in the organic phase containing a PLGA/PLA polymer to form a primary emulsion. This primary emulsion is atomized in a stream of heated air with a proper inlet/outlet temperature, and then the solvent evaporates instantaneously from the droplets formed to produce microspheres of particle size ranges from 1 to 100 µm depending on the atomizing conditions. The microspheres are collected from an airstream using a cyclone separator. Further, vacuum drying might be required to remove the residual solvents. The entire spray drying process can be operated in closed-loop configurations under aseptic conditions. Various spray drying process parameters, including inlet/outlet temperature, drying flow rate, feed rate, and atomization energy input, influence the physicochemical properties of PLGA/PLA microspheres and thus the stability and release profile of encapsulated proteins/peptides [94,95,96]. The proven reproducibility, control of particle size, and drug release properties of the spray dried microspheres are some of the advantages of the spray drying technique over other microencapsulation techniques. The spray drying processing conditions may be likely to induce stress (aggregation and denaturation), resulting in stability issues for microencapsulated proteins [97].

Although the inlet airflow temperature is high, the contact time between the hot air and sprayed droplets is limited. Therefore, when the sprayed droplets and hot airflow proceed through the chamber in the same direction, the droplets will absorb the heat, and their surface temperature will increase. However, in practice, the actual product temperature is about 15 to 25 °C lower than the outlet air temperature. Therefore, it is feasible to use the spray drying technique for the encapsulation of heat-sensitive molecules, such as peptides and proteins, by controlling the spray drying process parameters [98,99]. The processing parameters involved in the formation of primary emulsion in the double emulsion evaporation method may also influence the spray drying process in a similar manner. Since no outer solvent phase is used in the encapsulation of microspheres by the spray drying process, high entrapment efficiency is possible for hydrophilic molecules [100]. However, the microsphere formation process and the final characteristics of the microspheres can be tuned by selecting the appropriate solvent composition to adjust the feed drying kinetics and the solute precipitation rate. Recently, a three-fluid nozzle has been used to produce PLGA/PLA microspheres, where the aqueous phase (peptide/protein solution) and organic phase (PLGA/PLA) are fed through two separate channels into the drying chamber and then atomized into fine droplets. The two separate liquid channels can avoid the interfacial stresses of the aqueous and organic phases, and the feed rate ratio of two liquid channels is one critical parameter for this method [101,102]. The one-step, continuous spray drying process is easy to scale up, and the residual solvents in the spray dried microspheres is lower than the microspheres produced by the solvent evaporation method due to the heated airflow in the drying chamber [103].

## 4. Physicochemical Characteristics and Analytical Techniques of PLGA/PLA-Based LAI Microspheres

**Figure 5:**
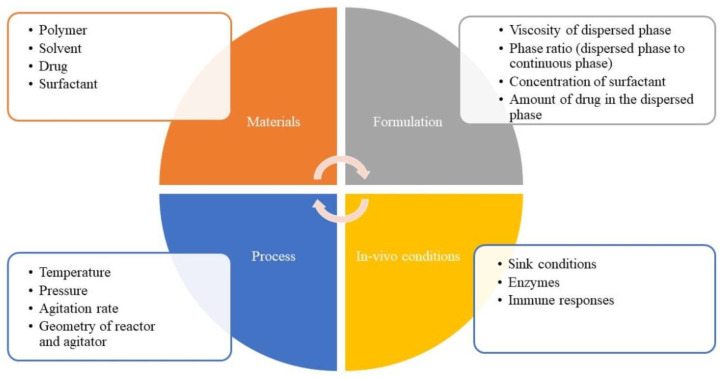
Different factors influencing the physicochemical properties of the PLGA/PLA-based LAI microspheres.

Complex LAI microsphere products have been used to deliver protein/peptide therapeutics over weeks to months in a controlled manner. The LAI microsphere products have complex formulation constituents and complicated manufacturing processing steps [22]. The physicochemical properties of PLGA microspheres are determined by several factors: type of polymer (polymer MW, monomer composition, and polymer functionalization), production method, processing and sterilization, drug and formulation parameters, and the presence of excipients such as stabilizers, surfactants, or osmotic agents. Small manufacturing changes can affect the physicochemical characteristics of microsphere drug products, which in turn influence the product in vitro and in vivo performance. Different factors affecting the properties of PLGA/PLA LAI microspheres are presented in Figure 5.

A variety of characterization techniques have been used to determine physicochemical characteristics and ensure consistency in manufacturing processing and product performance of protein/peptide loaded microspheres. These analytical techniques are used to determine the physicochemical characteristics of the microspheres, such as particle size and shape, polymer molecular weight, encapsulation efficiency, in vitro drug release, glass transition temperature, residual organic solvent, moisture content, and porosity. However, despite widespread use of microspheres, no specific guidance or standard method has been developed for in vitro release testing. Some of these physicochemical properties are determined during the microsphere development stage to guide formulation and process development, and some are tested for quality control purposes. A summary of various techniques for testing the physicochemical properties of microspheres is presented in Table 2.

### 4.1. In Vitro Release

In vitro release testing methods with reproducibility and good discriminatory ability are critical for both quality control purposes and to assist in product development. It is important to understand the release mechanisms and factors that affect the release rate in order to modify the drug release. For LAI PLGA/PLA microspheres, real-time release testing utilizes an extended period, which affects the product batch release time. Therefore, accelerated in vitro release methods that correlate with the real-time in vitro release of microsphere products are essential. Several factors, including temperature, pH, and presence of enzymes and surfactants, can expedite the rate of polymer hydration/degradation and drug diffusion, thereby accelerating drug release from PLGA microspheres [121,122]. There are four possible release mechanisms for drug molecules to be released from PLGA-based microspheres: (i) diffusion through water-filled pores, (ii) diffusion through the polymer, (iii) osmotic pumping, and (iv) polymer erosion. Of these, diffusion through water-filled pores is the most common if the encapsulated drugs used are large, hydrophilic biopharmaceuticals (e.g., proteins and peptides) [123].

During the in vitro release of PLGA-based LAI microspheres, water is absorbed by PLGA upon immersion in aqueous media or administration in vivo, and the volume occupied by the water inside the PLGA matrix creates pores. This porous connected network allows drug release as the number and size of water-filled pores increases in the polymer matrix [124]. Also, the scission of ester bonds (hydrolysis) and subsequent decrease in polymer MW occurs upon contact with the water. Hydrolysis creates acids, which catalyzes hydrolysis, causes heterogeneous degradation inside PLGA matrices, i.e., degradation of PLGA matrix at the center is faster than at the surface, and this outcome becomes more evident with increasing particle dimensions as the acid gradient increases [125,126,127]. This hydrolysis effect makes the polymer less hydrophobic with decreasing MW, and at a particular molecular weight (1100 Da), the oligomers become water soluble. Erosion (mass loss) of the polymer starts when the dissolved polymer degradation products are able to diffuse into the release medium. The dissolved polymer degradation products enhance the drug release in several ways (by catalyzing the hydrolysis due to its acidic nature, plasticizing the polymer (which decreases the polymer transport resistance due to an increase in the rate of water absorption), and increasing the osmolality inside the polymer matrix (which can be a driving force for water absorption) [128,129].

Due to the lack of compendial in vitro release methods, various in vitro release testing methods such as sample-and-separate, membrane dialysis, and continuous flow have been widely used for in vitro release testing of microsphere products [130]. Sample and separate methods can provide a direct and accurate assessment of in vitro drug release. However, inadequate agitation during release testing causes aggregation of microspheres and loss of the dosage form during sampling. In the case of the membrane dialysis methods, limited media volume is available inside the dialysis sacs, compromising the sink conditions when the drug is poorly soluble in the release media. USP apparatus 4 with well-defined geometry and hydrodynamics can minimize the microsphere aggregation while also avoiding microsphere loss during sampling, and has been demonstrated to be an appropriate in vitro release testing method for PLGA/PLA microspheres [105,130].

### 4.2. Particle Size

The particle size of PLGA/PLA microspheres influences the drug release behavior and injectability of the microspheres. Particle size varies with the different microsphere preparation techniques. The smaller particle size shows an enhanced initial burst release than the large size microspheres due to increased specific surface area. On the other hand, the polymer degradation rate is more prominent with the large microspheres compared with the small ones [18]. The erosion rate of PLGA polymer is also greater in the case of smaller sized microspheres compared to larger size microspheres. When comparing the particle size of microspheres manufactured via different preparation methods, particle size distribution is more meaningful than average particle size. Because microspheres with the same average particle size but different size distributions may exhibit different drug release profiles. To produce microspheres with desired particle size or size distribution, several factors need to be considered, which includes but are not limited to; polymer and surfactant type, the concentration of polymer in the organic phase, volume fraction of dispersed phase, stirring rate during hardening, homogenization speed, and the temperature during preparation [6,18].

### 4.3. Encapsulation Efficiency

Encapsulation efficiency is described as the amount of protein/peptide encapsulated into microspheres following preparation. The physical and chemical properties of the polymer, the solvent used in the encapsulation process, and drug–polymer interactions can influence the encapsulation efficiency of proteins/peptides. It has been reported that microsphere preparation temperature in the solvent evaporation method is likely to affect the encapsulation efficiency. At a low preparation temperature, the immiscibility between the polymer phase and the water phase is increased, leading to the rapid formation of the microsphere wall. On the other hand, at a higher preparation temperature, the solvent evaporation rate is increased, resulting in the quicker formation of the microsphere similar to that observed at the low preparation temperature [6]. It is noteworthy that the potency and the corresponding drug loading efficiencies play an important role in the long-term release of peptide/protein drugs. For a highly potent peptide/protein, a low drug loading efficiency might be sufficient to achieve the desired pharmacokinetic profile. In contrast, for a peptide/protein drug with low potency, a sufficiently high drug loading efficiency is required to obtain prolonged pharmacokinetic exposure [18].

### 4.4. Porosity

Porosity, either on the surface or in the internal polymeric matrix, significantly affects the drug release profiles of the microspheres. The pore size of microspheres can be modulated by selecting the appropriate solvent. The organic solvent with lower volatility produces large pores, whereas a more volatile solvent gives rise to small pores [131]. The polymer concentration also influences the microsphere’s porosity. The porosity degree of particles reduces with increasing polymer concentration without significantly affecting the mean diameter of microspheres [132]. Microspheres with a high porosity results in undesirable initial burst release, leading to the release of drug over a relatively short period. During the solvent evaporation process, the water soluble protein/peptides will tend to diffuse into the external aqueous phase, creating a channel on the microsphere surface as well as the internal polymeric matrix. Thus, the encapsulated drug will quickly leach out through the pores upon contact of the microspheres with an aqueous environment [6].

### 4.5. Glass Transition Temperature

The Tg of the PLGA microspheres is closely related to the amount of residual solvent in the formulation. While preparing PLGA microspheres, several formulation and process parameters influence the Tg of the polymer, which include but are not limited to PLGA type, drug physicochemical characteristics, residual solvent (s), drying rate, and post-treatment [133]. Typically, Tg of PLGA polymer decreases with a reduction in polymer molecular weight or a reduction in lactic acid content [40]. It has been reported that PLGA microspheres are most commonly prepared by the emulsion based method, where the polymer is dissolved in organic solvent and becomes hardened once the solvent is extracted or evaporated [133]. Therefore, the type of organic solvent used and its concentration is one most important factors affecting the Tg of the PLGA microspheres [134]. The drug release profile of PLGA microsphere formulations can be controlled by manipulating the parameters influencing the Tg of the formulation. Depending on the polymer properties, the drug itself can lower the Tg of the microsphere formulation through its plasticization effect, which occurs from the interaction of the drug with PLGA polymer [135,136]. However, drugs such as leuprorelin acetate show an anti-plasticizing effect, resulting in an increase in Tg [137].

### 4.6. Particle Morphology

The particle morphology of PLGA microspheres can influence the product performance, particularly the in vitro release profile. A modification in the manufacturing process is known to affect particle morphology [138]. It has been reported that microspheres prepared at low stirring rate show a spherical, smooth surface morphology with a relatively uniform size [139]. The concentration or viscosity of PLGA and its copolymer ratio can influence particle morphology. With the higher concentration (4 wt% polymer solutions) of PLGA, the particles formed were larger and the outer surface of the particles stayed smooth. The solutions from a PLGA copolymer ratio of 50:50 (lactide to glycolide) produced spherical particles, while solutions from lactide to glycolide of 75:25 show elongated or irregular particles [140]. In the emulsion solvent evaporation method, the morphology of microspheres is influenced by the rate of polymer precipitation during the solvent removal step. A slow evaporation/removal of the organic solvent produces a smooth surface due to the slow precipitation of the polymer. On the other hand, the quick removal of organic solvent gives a porous surface. When using ethyl acetate in the organic phase, PLGA microspheres express a rough surface owing to its high boiling temperature, which does not allow for a complete solidification process [141].

## 5. Recent Progress in PLGA-Based LAI Microspheres

Recent progress in the area of PLGA long-acting injectable formulations mainly includes: demonstrating the influence of raw materials and manufacturing variables on the performance of LAI microspheres; exploring biorelevant in vitro–in vivo correlations (IVIVCs) for biodegradable injectable PLGA microspheres; obtaining a better understanding of the impact of properties of PLGA polymers on product performance; developing modeling tools to facilitate the development of generic LAI formulation development as well as bioequivalence guidances for LAI formulations; investigating potential peptide PLGA interactions during product manufacturing and use; and developing an analytical method for separating PLGA polymers. Recent studies aimed to determine the qualitative sameness of PLGA polymers because altered PLGA characteristics have been recognized as a critical factor that may cause performance variation in PLGA microsphere drug products. Similar PLGA polymers from different sources may have different physicochemical properties such as inherent viscosity, Mw, Tg, and blockiness. This could significantly impact physicochemical properties (such as the particle size distribution and the internal microstructure) and consequently the release characteristics of PLGA LAI microspheres. Bo Wan et al. [142] evaluated minor differences in the physicochemical properties of PLGA polymers from various sources, differences in the physicochemical properties and in vitro release of leuprolide acetate microspheres have been investigated. The findings suggest that differences in polymer sources have a considerable impact on the sameness of physicochemical properties and the therapeutic performance of long-acting PLGA microspheres. The basic knowledge of polymer characteristics gained will be crucial in the creation of quality control measures and future regulatory guidance on the evaluation of LAI PLGA microspheres.

IVIVC can be used to predict the in vivo burst release based on the in vitro burst release of microsphere formulations, which helps to predict the in vivo performance of microsphere formulation(s) with no or minimal burst release [143]. It has been challenging to establish an IVIVC for complex parenteral microsphere formulations due to a combination of factors, including their unique traits (such as multiphase drug release profiles) but also the lack of a standard/compendial in vitro release testing method, which can mimic and predict their in vivo performance to the maximum extent possible [61,115]. There is a scarcity of the establishment of IVIVC for complex parenteral microsphere drug products. Recent research has shown that a reliable Level A IVIVC can be developed for compositionally equivalent LAI PLGA microspheres with manufacturing differences [144,145]. Diane J Burgess and her team [61] reported in aIVIVC of parenteral naltrexone loaded polymeric microspheres. Three naltrexone PLGA microspheres with similar compositions but different fabrication methods were prepared. A previously designed USP apparatus 4 method was used to determine the in vitro release properties of the produced naltrexone microsphere formulations and the reference listed drug (RLD) product Vivitrol^®^. The in vitro release profiles of naltrexone microspheres were compared to the pharmacokinetic profiles of the microspheres using a rabbit model to develop an IVIVC and evaluate its prediction. The findings showed that the established USP 4 approach was capable of detecting manufacturing process-related performance changes, as well as forecasting the in vivo performance of naltrexone microspheres in the tested animal model. The bi-phasic and tri-phasic release characteristics of naltrexone microspheres, with varied burst release and lag phase, are a significant difference. The evolution of IVIVCs is influenced by these differences in release profiles.

In recent years, considerable research has been conducted to develop modeling tools to facilitate the development of generic LAI formulation development as well as bioequivalence guidance for LAI formulations. A tool for developing models appropriate for characterizing the complex absorption process and the pharmacokinetic time course of LAI formulations is challenging. The plasma concentration–time profiles following administration of LAI PLGA formulations are often irregular and cannot be interpreted easily with conventional models based on first-order absorption kinetics and lag time. Sam N. Rothstein’s team [146,147] demonstrated a simple, deterministic model that can accurately forecast the release of a wide range of agents encapsulated in bulk biodegradable PLGA polymer matrices. There is a gap in the current scientific understanding of how different formulations and raw material variables used to manufacture peptide encapsulated PLGA microspheres lead to different levels of peptide–polymer interactions and peptide acylation during encapsulation, storage, and release in vitro and in vivo. Unpredictable acylation due to peptide–polymer interaction could lead to variations in pharmacokinetics and a loss of bioequivalence of generic long-acting release PLGA formulations, which may lead to differences in safety and efficacy [148]. Cationic peptides are known to bind efficiently to PLGAs with a carboxylic acid (COOH) end group, which makes developing PLGA-based peptide therapeutic delivery systems difficult. This interaction is thought to be a critical step in the peptide acylation process in PLGA-based formulations, as it affects microencapsulation and release [148]. Schwendeman and his group [78,148,149,150] investigated the thermodynamics of peptide−PLGA binding in dimethyl sulfoxide using a model cationic octapeptide, octreotide, utilizing the nano isothermal titration calorimetry method. Results showed that the extent of the interaction with the octreotide was exclusively reliant on the availability of the acid end group of the PLGA. Furthermore, they concluded that understanding the underlying driving force of peptide–PLGA binding can help formulate formulations that prevent the peptide acylation precursor step in PLGA-based long-acting release formulations and discover differences between PLGA/peptide formulations.

PLGA is the key component of LAI drug products responsible for providing controlled and sustained drug release. It is important to determine key PLGA characteristics to ensure comparable product performance because of the impact PLGA has on drug release kinetics. John Garner et al. [113] developed a protocol for determining the key properties of PLGA, the L:G ratio, polymer molecular weight distribution, and end-cap in clinical formulations containing PLGA microparticles. The protocol outlines a procedure for isolating PLGA from microparticles by eliminating additional excipients and active pharmaceutical ingredients as well as analysis procedures for identifying the primary parameters. This protocol will be valuable in developing PLGA-based long-acting therapeutic products because the features of individual PLGA in clinically utilized formulations are not easily available. Furthermore, the ability to determine key PLGA properties will aid in the formulation of generic PLGA depot microsphere formulations.

## 6. Conclusions

Although PLGA/PLA is the most widely used biodegradable and biocompatible polymer in LAI microsphere formulations, the limitations, including incomplete drug release profile and burst release, should be considered while developing an LAI depot of PLGA/PLA microspheres. Among the three conventional manufacturing techniques (emulsion–solvent evaporation, coacervation, and spray drying), emulsion–solvent evaporation is the most applicable method in clinical LAI microspheres. However, in the emulsion–solvent evaporation method, several process parameters such as polymer concentration, phase ratio, surfactant concentration, protein loading, and intensity of homogenization should be optimized because these parameters can affect the characteristics of microspheres such as particle size, release profile, and encapsulation efficiency. By modifying the PLGA characteristics, it is possible to produce a microsphere formulation with desired properties and physiological behavior. Further, it is important to understand and select appropriate processing conditions to guarantee the desired physicochemical characteristics of the LAI microspheres. The high complexity of the manufacturing process, absence of adequate standards, and product-specific regulations hindered the progress of developing generic LAI depots of PLGA/PLA microspheres. Although FDA guidance on LAI microspheres is not complete, the information available from the existing literature might be informative and instructive to bring LAI PLGA/PLA microsphere products to the market in the near future.

## Figures and Tables

**Table 1:** FDA approved commercial PLGA/PLA-based long-acting injectable microspheres containing proteins and peptides [18,19].

Clinical Products	Active Agent	Polymer	Encapsulation Technique	Duration	Company
**Sandostatin^®^ LAR**	Octreotide acetate	PLGA glucose	Coacervation	4 weeks	Novartis
**Signifor^®^ LAR**	Pasireotide pamoate	PLGA	Emulsion solvent evaporation	4 weeks	Novartis
**Somatuline^®^** **Depot**	Lanreotide acetate	PLGA	Spray drying	4 weeks	Ipsen
**Lupron Depot^®^**	Leuprolide acetate	PLGA/PLA	Emulsion solvent evaporation	I.M/4, 12,	Takeda
**Trelstar^®^**	Triptorelin pamoate	PLGA	Spray drying or coacervation	I.M./4, 12, 24 weeks	Allergan
**Bydureon^®^**	Exenatide	PLGA sucrose	Coacervation	Weekly	Astra–Zeneca

**Table 2:** Summary of analytical techniques used for the characterization of protein /peptide loaded PLGA/PLA-based LAI microspheres [15,18,36,104,105,106,107,108,109,110,111,112,113,114,115,116,117,118,119,120].

Technique	Principle	Purpose	Comments
Gel permeation chromatography	Species separation according to their MW, high MW species being eluted first.	Measure the MW of polymers.	Evaluate the degradation behavior of PLGA/PLA in microspheres during in vitro release by measuring the MW of PLGA/PLA at different time points.
Nuclear magnetic resonance	A small chemical shift in the spectrum arises as variations in the magnetic field occur due to the interaction of orbiting electrons with the nucleus in varying chemical environments.	Characterize the polymer properties such as the ratio of lactic and glycolic acid units and end-cap group.	The ratio of lactic and glycolic units and the end-cap group of PLGA can be determined by ^1^H NMR and ^13^C NMR, respectively.
Differential scanning calorimeter	Measure enthalpy changes due to changes in the physical and chemical properties as a function of temperature.	Characterize the solid-state of the polymers.	Determine the glass Tg of the PLGA in the microspheres.
Laser diffraction	Measure the amount of light blocked when a particle gets in front of the beam, as particles pass as a single file through the detector.	Determines the particle size and size distribution of microspheres.	The size distribution is described as span value, calculated as D_90_, D_50_, and D_10_. Pre-ultrasonication is required because the agglomeration of small particles may disturb the measurement. Analysis is fast, stable, and accurate.
High performance liquid chromatography	Separation of components in a liquid mixture. A liquid sample is injected into a mobile phase flowing through a column packed with a separation medium.	Characterize and quantify proteins/ peptides and their chemical degradation.	Determines the important properties of the microspheres such as drug load and drug release on an established standard curve.
Gas chromatography	Separation of the components present in the mixture based on the partition between the gaseous mobile phase and liquid stationary phase.	Evaluate the residual solvent content to assure that it is within the acceptance limit.	Most appropriate method due to their various advantages such as lowest detection limits, ease of sample preparation, and specificity compared to other analytical techniques such as thermo-gravimetric analysis and spectrometric methods.
Scanning electron microscopy (SEM)	Accelerated electrons in an SEM carry significant amounts of kinetic energy, and this energy is dissipated as a variety of signals produced by electron–sample interactions when the incident electrons are decelerated in the solid sample.	Detect the size, surface structure, and shape of microspheres.	Standard method to obtain information on the microstructure of the microspheres.
Sodium dodecyl sulfate–polyacrylamide gel electrophoresis	Molecules are separated according to the length and charge of the polypeptide chain.	Determines the structural integrity of proteins/peptides.	Identify the MW change of proteins/peptides after encapsulation. The aggregation or structural integrity of the proteins/peptides can be measured to some extent.
Fourier transform infrared	Explores bonds vibrations in order to provide a second-derivative spectrum.	Detects the secondary structural integrity of peptides/proteins and determines the interaction between the polymer and the encapsulated drug.	Detect the interaction between proteins/peptides and polymer matrix and identify the structural change of proteins/peptides after encapsulation.
USP apparatus IV	The flow-through method using the official USP IV apparatus operated in open-loop mode is capable of maintaining a continuous flow of fresh dissolution medium, thus maintaining infinite sink conditions. The microspheres sample are mixed with glass beads in a sandwich manner.	USP apparatus IV is the recommended compendial method for in vitro release testing of microspheres.	Compared to conventional in vitro release methods (sample and separate and USP apparatus II), USP apparatus IV method provides the highest cumulative release and lowest variation in data, offers easy maintenance of sink conditions, easy change of media and media volume can be easily adjusted during the test.
Mercury porosimeter	Measures both the pressure and volume of mercury taken up by a porous material.	Determines the porosity of microspheres.	Measure the total intrusion volume, total pore area, and porosity.

## Data Availability

Not applicable.
